# A Quantitative Method for Acesulfame K Using the Taste Sensor

**DOI:** 10.3390/s20020400

**Published:** 2020-01-10

**Authors:** Yuanchang Liu, Xiao Wu, Yusuke Tahara, Hidekazu Ikezaki, Kiyoshi Toko

**Affiliations:** 1 Graduate School of Information Science and Electrical Engineering, Kyushu University, 744 Motooka, Nishi-ku, Fukuoka 819-0395, Japan; 2 Research and Development Center for Five-Sense Devices, Kyushu University, 744 Motooka, Nishi-ku, Fukuoka 819-0395, Japan; wu.xiao@nbelab.ed.kyushu-u.ac.jp (X.W.); tahara.yusuke.494@m.kyushu-u.ac.jp (Y.T.); toko@ed.kyushu-u.ac.jp (K.T.); 3 Intelligent Sensor Technology, Inc., 5-1-1 Onna, Atsugi-shi, Kanagawa 243-0032, Japan; ikezaki.hidekazu@insent.co.jp; 4 Institute for Advanced Study, Kyushu University, 744 Motooka, Nishi-ku, Fukuoka 819-0395, Japan

**Keywords:** sweetness sensor, taste sensor, lipid polymer membrane, acesulfame K, high-potency sweeteners

## Abstract

We have developed a method to quantify the sweetness of negatively charged high-potency sweeteners coexisting with other taste substances. This kind of sweetness sensor uses lipid polymer membranes as the taste-sensing part. Two types of outputs have been defined in the measurement of the taste sensor: one is the relative value and the other is the CPA (the change in membrane potential caused by adsorption) value. The CPA value shows a good selectivity for high-potency sweeteners. On the other hand, the relative value is several times higher than the CPA value, but the relative value is influenced by salty substances. In order to obtain both high sensitivity and selectivity, we established a model for predicting the concentration of sweeteners with a nonlinear regression analysis method using the relative values of both the sweetness sensor and the saltiness sensor. The analysis results showed good correlations with the estimated concentration of acesulfame potassium coexisting with salty substances, as represented by *R*^2^ = 0.99. This model can correspond well to the prediction of acesulfame K in a concentration of 0.2–0.7 mM, which is commonly used in food and beverages. The results obtained in this paper suggest that this method is useful for the evaluation of acesulfame K using the taste sensors.

## 1. Introduction

Sight, hearing, touch, taste and smell are the five basic senses of human beings. With these senses, we can explore the world in multiple dimensions—not only to survive, but also to live a better life [1]. There are five basic tastes, which are sour, sweet, bitter, salty and umami. Different tastes often represent different characteristics of food. Sweetness indicates that the food contains sugar and can provide energy. Bitterness often represents the presence of toxic substances, while saltiness represents minerals [2].

With the development of the chemical industry and the food manufacturing industry, the correspondence between taste and food ingredients has become complicated. Over the past 20 years, increasing kinds of high-intensity, low-calorie sweeteners (e.g., acesulfame K and aspartame) have been discovered or synthesized [3,4]. The foods made with those sweeteners share the same sweetness as traditional sugars (e.g., sucrose) while containing almost no calories.

In recent years, high-potency sweeteners have become very popular in the beverage and medical industries [5]. High-potency sweeteners are dozens of times sweeter than sucrose and have almost no calories [4]. Increasing numbers of low-calorie or sugar-free foods are made with high-potency sweeteners. These sugar-free foods can also be purchased by diabetics. For manufacturers, using high-potency sweeteners can save costs compared to using sucrose [6].

High-potency sweeteners can be dividing into three types according to their different charging states in solution: positively charged (e.g., aspartame), negatively charged (e.g., acesulfame K and saccharin Na), and uncharged (e.g., stevia extract and sucralose). The range of high-potency sweeteners commonly contained in beverages is about 0.2 to 0.7 mM for acesulfame K and about 0.1 to 0.5 mM for saccharin Na, which are very low concentrations compared to sucrose [7]. Some high-potency sweeteners will have a bitter taste that is unpleasant when entering the mouth for a while at a high concentration [8]. Manufacturers often use a mixture of sweeteners to enhance the sweetness of products in order to achieve enough sweetness while avoiding the residue of a bitter taste [6,7]. Therefore, high-potency sweeteners tend to maintain a very low concentration range in beverages.

With the widespread use of high-potency sweeteners, there is a great need to evaluate the sweetness of products containing high-potency sweeteners. Due to the wide variety of high-potency sweeteners and their low concentration in the products, there is currently a need for a low-cost and rapid evaluation method. In the development process, the products are often tasted by specially trained inspectors using a sensory test. Sometimes there are several people in the food development process, forming a team called a “focus group” for sensory analysis [9,10]. They evaluate foods and record the results by direct tasting.

In the food and beverage industry, the brix refractometer is used to measure the sugar content of an aqueous solution as degrees Brix (symbol °Bx). However, the degrees Brix score is based on the total soluble solids content (TSS) [11,12]. The other solutes in the solution will also affect the value of the Brix. Therefore, degrees Brix does not directly correspond to the intensity of sweetness [13].

HPLC (high-performance liquid chromatography) is used for the quantitative analysis of liquid samples and is also commonly used in the study of sweeteners [14,15]. While HPLC is used to analyze the content of different sweeteners in the solution, it hardly comprehensively evaluates the sweetness of the solution. In addition, HPLC is very expensive and cumbersome for general food quality control.

There are many electronic tongues (e-tongues) in the world; the taste sensor developed by Professor Toko in Kyushu University is a kind of e-tongue. The taste sensing system TS-5000Z (Intelligent Sensor Technology, Inc., Astsugi-shi, Japan) was developed for many years [16]. Using a lipid polymer membrane as the sensing part, the taste sensor is designed to have a characteristic called global selectivity as a goal. The global selectivity means that the taste sensor can respond to each taste quality by classifying enormous kinds of chemical substances into five kinds of groups (sourness, saltiness, bitterness, sweetness, and umami) in accordance with the physicochemical properties of each basic taste quality, such as hydrophobicity and iconicity [17,18,19,20]. The taste sensor is designed and developed with global selectivity to distinguish different tastes, which is its main difference from other e-tongues. Furthermore, the taste sensor can evaluate tastes using fewer electrodes than other kinds of e-tongues. In recent years, the taste sensor has been widely used for the quantification of taste in many applications, such as tea [21,22], milk [23], rice [24], pork [25], table salt [26], and so on [16].

According to the Weber–Fechner law, the amount of human senses is proportional to the logarithm of stimulus strength when subjected to moderate stimulation [27,28]. It was also found that the response of the taste sensor is proportional to the logarithm of the concentration of the taste sample [29]. Therefore, it is considered to be possible to reflect the sense of taste of human beings using the responses of a taste sensor.

We have been working on the development of a taste sensor for high-potency sweeteners for several years. This kind of sweetness sensor has a good selectivity to high-potency sweeteners at the CPA value, because the CPA value represents the potential change caused by the hydrophobic interaction. However, the CPA value is usually lower than another kind of output, called the relative value, which is caused by both hydrophobic interaction and electrostatic interaction [22,30]. On the other hand, the relative value of high-potency sweeteners is several times larger than the CPA value, but the sweetness sensor may respond to other interfering substances by electrostatic interaction. If there is no interfering substance or the influence of the interfering substances can be removed, the relative value showing a higher sensitivity rather than the CPA value can be used to evaluate high-potency sweeteners. This is very valuable for the measuring of beverages which usually contain high-potency sweeteners and interfering substances at the same time. Furthermore, the objective of this research is to confirm and eliminate the influence derived from the interfering substances and correctly quantify high-potency sweeteners in mixture solutions.

In this study, we have confirmed the selectivity of sweetness sensors for different basic taste substances, and we have tried to establish an analytical model to evaluate high-potency sweeteners in mixed solutions containing interfering substances.

## 2. Experiment

### 2.1. Chemicals

TDAB (tetradodecylammonium bromide) was purchased from Sigma-Aldrich Co., Ltd. TDA (1-Hexadecanol) was purchased from Tokyo Chemical Industry Co., Ltd., Tokyo, Japan. NPOE (2-nitrophenyl octyl ether) was purchased from Sigma-Aldrich Co., Ltd. PVC (polyvinyl chloride) was purchased from Sigma-Aldrich Co., Ltd. NaCl was purchased from Kanto Chemical Co., Inc., Tokyo, Japan. Acesulfame K was purchased from Tokyo Chemical Industry Co., Ltd., Tokyo, Japan. TDAB and TDA were used as lipids. NPOE and DOPP were used as plasticizers. PVC was used as a support material for the lipid polymer membrane. NaCl was used as a salty substance. Acesulfame K was used as a representative substance of negatively charged high-potency sweeteners.

### 2.2. Lipid Polymer Membrane

**Figure 1:**
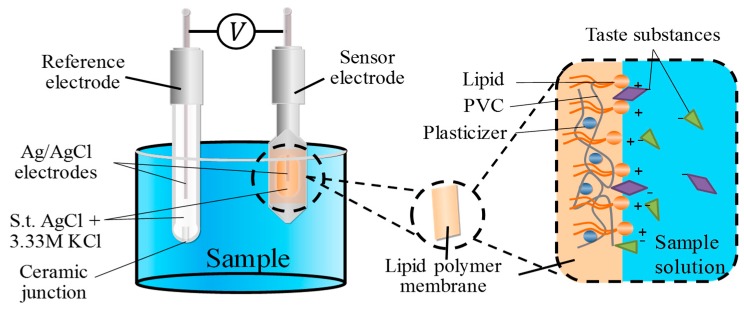
Membrane structure of taste sensor. PVC: polyvinyl chloride.

A taste sensor which adopts a lipid polymer membrane as a sensing part was used in this study. The lipid polymer membrane consists of a lipid, plasticizer and PVC [16]. The membrane can be charged in the solution due to the ionization of the lipid. The lipid content also determines the hydrophobicity of the membrane. The plasticizer is used to adjust the flexibility and hydrophobicity of the membrane. PVC is used as a supporting material. As shown in Figure 1, when the lipid polymer membrane is immersed in solutions, the hydrophilic group of the lipid can be ionized to charge the surface of the membrane [16,17], and then the lipid polymer membrane can attract the corresponding taste substances in solutions due to the electrostatic interaction and hydrophobic interaction. As a result, the membrane potential changes. Then, the intensity of the taste can be evaluated. In this study, the sweetness sensor for high-potency sweeteners consists of TDAB, NPOE and PVC, which we developed in a previous study [29]. The saltiness sensor consists of TDAB, TDA, DOPP and PVC [18].

### 2.3. Taste Sensor

The taste sensing system TS-5000Z (Intelligent Sensor Technology, Inc.) was used in this study. This system is equipped with different sensors for each taste. In a broad sense, the taste sensor is a kind of electronic tongue. However, there is a difference from other sensors: the taste sensor is developed with the goal of global selectivity [17]. Global selectivity means that each sensor can respond to a certain taste quality selectively [18]. By adjusting the composition and content of the lipid and plasticizer, sensors for different tastes are made. While evaluating the taste using a taste sensor, a working electrode and reference electrode are used. As shown in Figure 1, the working electrode is composed of an Ag/AgCl electrode and a probe with a lipid polymer membrane. The reference electrode is composed of an Ag/AgCl electrode and a glass tube with a ceramic junction at the bottom. Both the working electrode and the reference electrode are filled with 3.33 M KCl and saturated AgCl solution as an internal liquid. The sweetness sensor is developed by our research group. The saltiness sensor, called CT0, is from Intelligent Sensor Technology, Inc. In this study, both the sweetness sensor and the saltiness sensor were used to evaluate the sweetness of sample solutions coexisting with salty substances.

### 2.4. Measurement Process of Taste Sensor

**Figure 2:**
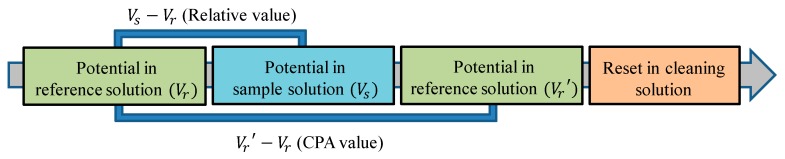
The measurement procedure of the taste sensor. CPA: change in membrane potential due to absorption.

As shown in Figure 2, the following steps are executed when measuring samples. First, sensors were immersed in the reference solution, and the membrane potential between the working electrode and reference electrode was measured as *Vr*. The composition of the reference solution was 0.3 mM tartaric acid and 30 mM KCl. After moving the sensor into the sample solution, the taste substances were attracted to the sensor membrane surface due to electrostatic interactions and hydrophobic interactions, as mentioned above in Section 2.2. The membrane potential between the electrodes in the sample was Vs. After that, the electrodes were immersed in the reference solution again and gently washed. In this process, hydrophilic substances were washed away, and the membrane potential decreased to *Vr*′. Finally, the electrodes were cleaned with an alcohol-based cleaning solution. Furthermore, *Vs*–*Vr* was defined as the relative value, and *Vr*′–*Vr* was defined as the CPA value. CPA stands for a change in the membrane potential caused by adsorption. On the other hand, the relative value was the change of membrane potential caused by both the electrostatic interaction and hydrophobic interaction [16,18,31].

### 2.5. Response Characteristics of Sweetness Sensor and Saltiness Sensor

Increasing numbers of sweeteners have been developed in recent years. Because the commonality of sweeteners has not yet been elucidated, it is still difficult to evaluate all the sweet substances with only one sensor. In the development of taste sensors, we have divided the sweeteners into three types: there are positively charged sweeteners (e.g., aspartame), negatively charged sweeteners (e.g., acesulfame K and saccharin Na) and uncharged sweeteners (e.g., glucose and sucralose). Furthermore, we developed the corresponding sweetness sensors [16,30,32]. One of the sweetness sensors is for negatively charged high-potency sweeteners such as acesulfame K and saccharin Na. The CPA value of this sweetness sensor shows a good selectivity for negatively charged high-potency sweeteners and it is not influenced by bitterness, sourness, umami or salty substances. Furthermore, the sweetness sensor shows a good concentration dependence for acesulfame K and saccharin Na [29]. Moreover, this sweetness sensor is different from the previous sensor which uses PTEH (phosphoric acid tris(2-ethylhexyl) ester) as a plasticizer. By adjusting the amount of lipid and changing the plasticizer to NPOE, this sensor shows no response to astringent substances and shows sensitivity to negatively charged high-potency sweeteners at the same level [32].

When measuring high-potency sweetener samples, the relative value is several times higher than the CPA value. Therefore, in order to improve the evaluation accuracy, the relative value was adopted. However, the sweetness sensor may respond to other taste substances caused by electrostatic interaction in the relative value. In order to find out the other interfering tastes causing the relative value, it is necessary to investigate the response characteristics of the sweetness sensor and the sensor for interfering taste to the basic tastes. The samples of various taste substances were prepared as shown in Table 1. Depending on the state of charge in the solution, positively and negatively charged sweeteners were used. All the samples are shown in Table 1.

The concentration of samples was determined by taking into account the amount of sweetener in the commercial beverage. Although the sweetness sensor responds to both acesulfame K and saccharin Na, we use acesulfame K as a representative substance in this study. The concentration of acesulfame K in the commercial beverage is about 0.2–0.7 mM [7]. The NaCl concentration as an interfering substance was added at four concentrations of 1, 10, 30, and 50 mM. The acesulfame K solution was prepared at four concentrations of 0.1, 0.25, 0.5, and 1 mM. The reference solution (0.3 mM tartaric acid + 30 mM KCl) was used as a vehicle for all the above samples.

### 2.6. Regression Analysis Model for Evaluating Sweetness

In order to evaluate the sweetness of acesulfame K in the mixture solutions with the interfering taste substances, a regression analysis was adopted using the sensor responses of both the sweetness sensor and the sensor for the interfering taste quality. Since there are no other substances that affect the response of sweetness and saltiness sensors, the sweetness can be evaluated using a data analysis method according to the responses of taste sensors. Thus, an analytical model can be used to evaluate the sweetness of acesulfame K in mixture solutions. As mixture solutions, a total of 16 mixed samples of acesulfame K (0, 0.25, 0.5, 1 mM) and the interfering substance NaCl (0, 10, 30, 50 mM) were prepared. The relative values of the 16 mixed samples were measured using both the sweetness sensor and the saltiness sensor.

For human senses, according to the Weber–Fechner law, the amount of sensation is logarithmically related to the amount of stimulation [19,20]. For the taste sensor, the relationship between the response of sensor and concentration of taste substances can be approximated by the *a* logarithmic function [29]. To describe the relationship between variables simply and effectively, the *a* logarithmic function is used to establish the relationship between the concentration of the taste substance and the relative value, as given by Equations (1) and (2).

(1) F(x,y)=a·log(p·x+h·y)

(2) G(x,y)=b·log(q·x+i·y)

where *F* is the relative value response of the sweetness sensor, *G* is the relative value response of the saltiness sensor, *x* is the concentration of acesulfame K, *y* is the concentration of NaCl, and *a*, *b*, *p*, *q*, *h* and *i* are numerical constants.

Thus, the relationship of the relative value and the concentration of the taste substance can be expressed in Equations (3) and (4):

(3) $$x=j\cdot \exp\left(\frac{F}{a}\right)-k\cdot \exp\left(\frac{G}{b}\right)$$

(4) $$y=m\cdot \exp\left(\frac{F}{a}\right)-n\cdot \exp\left(\frac{G}{b}\right)$$

where *j*, *k*, *m* and *n* are numerical constants defined by Equation (5) in order.

(5) $$j=\frac{h}{qh-ip},\;k=\frac{i}{qh-ip},\;m=\frac{q}{qh-ip},\;n=\frac{p}{qh-ip}.$$

The sensor responses *F* and *G* are affected by both saltiness and sweetness. Taking the relationship between *F* and *G* into account, the interaction terms of Equations (6) and (7) are used. As we know, adding interaction terms is believed to be able to maximize the inter-relationship among parameters and is also used in regression analysis in other sensor research [33]. These two interaction terms will only take effect if *F* is not equal to *G*. It is more convenient to modify the prediction model by adjusting the parameters of interaction terms when there are differences in the responses of the two sensors.

(6) $$c\cdot \left(\exp\left(\frac{F-G}{e}\right)-1\right)$$

(7) $$d\cdot \left(\exp\left(\frac{F-G}{f}\right)-1\right)$$

where *c*, *d*, *e* and *f* are numerical constants.

Therefore, from Equations (3)–(7), we obtain the prediction model of Equations (8) and (9).

Concentration of acesulfame K:

(8) $$x=j\cdot \exp\left(\frac{F}{a}\right)+k\cdot \exp\left(\frac{G}{b}\right)+c\cdot \left(\exp\left(\frac{F-G}{e}\right)-1\right),$$

Concentration of NaCl:

(9) $$y=m\cdot \exp\left(\frac{F}{a}\right)+n\cdot \exp\left(\frac{G}{b}\right)+d\cdot \left(\exp\left(\frac{F-G}{f}\right)-1\right),$$

## 3. Results and Discussion

### 3.1. Response Characteristics of Sweetness Sensor and Saltiness Sensor

**Figure 3:**
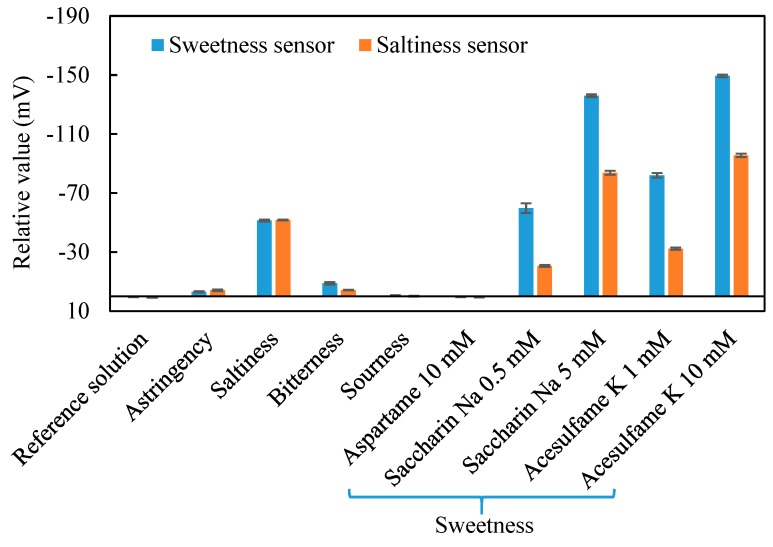
Selectivity of sweetness sensor and saltiness sensor by using relative values.

The response of the sweetness sensor and saltiness sensor to different kinds of taste substances is shown in Figure 3. Both the sensors respond to the saltiness (NaCl) sample and sweetness (acesulfame K) sample in the relative values. For saltiness samples, both the sensors exhibited an equivalent response of about −50 mV. The sweetness sensor shows higher responses than the saltiness sensor for the same concentration of high-potency sweeteners. The sweetness sensor was able to achieve a response of about −60 mV for 0.5 mM saccharin Na and about −80 mV for 1 mM acesulfame K. Both sweetness and saltiness sensors did not respond to astringency, bitterness or sourness. The concentration of each taste substance is ten times the human threshold, which is high enough compared to samples in practical applications. As a result, the sensor shows almost a negligible response to the basic taste samples (shown in Table 1), indicating that it is less likely to be affected in practical applications. Therefore, the sweetness of the sweetener can be evaluated if the influence of the salty substance is removed.

**Figure 4:**
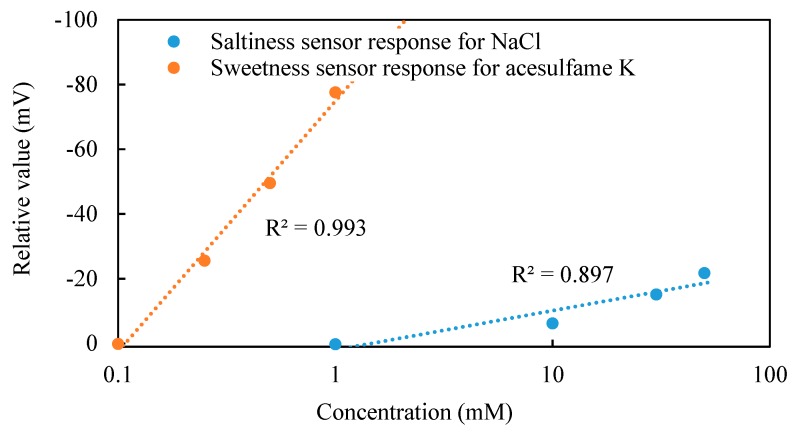
Relative values of the sweetness sensor and saltiness sensor.

Figure 4 shows the relative values of the sweetness sensor for different concentrations of acesulfame K, and the saltiness sensor for different concentrations of NaCl. Relative values of the sweetness sensor changed in a concentration-dependent manner and can be well approximated with a logarithmic function to the concentration of acesulfame K. Similarly, the relative values of the saltiness sensor also showed a dependence on the concentration of NaCl. Therefore, it is suggested that the logarithmic function can be used to represent the relationship between the relative value and the concentration of taste substances.

### 3.2. Regression Analysis Model for Evaluating Sweetness

**Figure 5:**
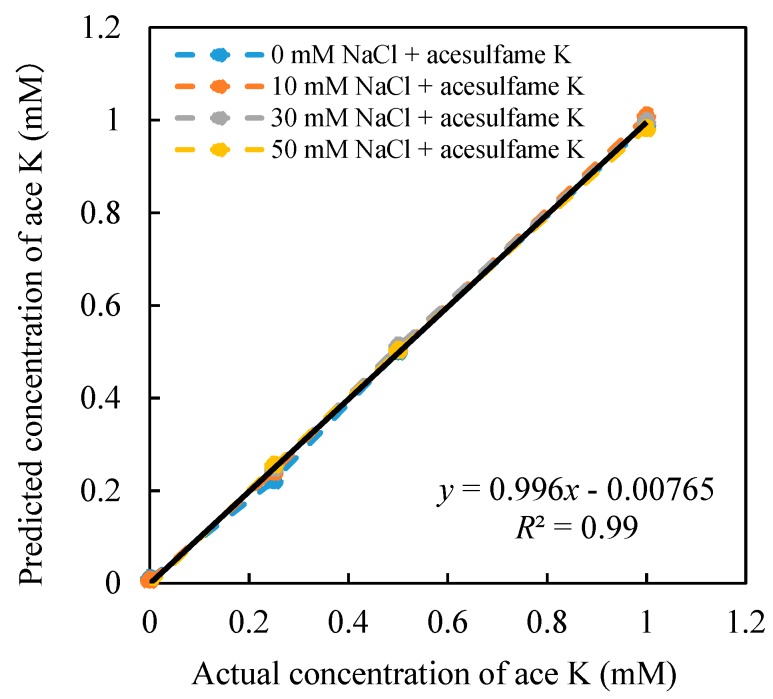
Relationship between the estimated concentration and the actual concentration of acesulfame K (ace K).

**Figure 6:**
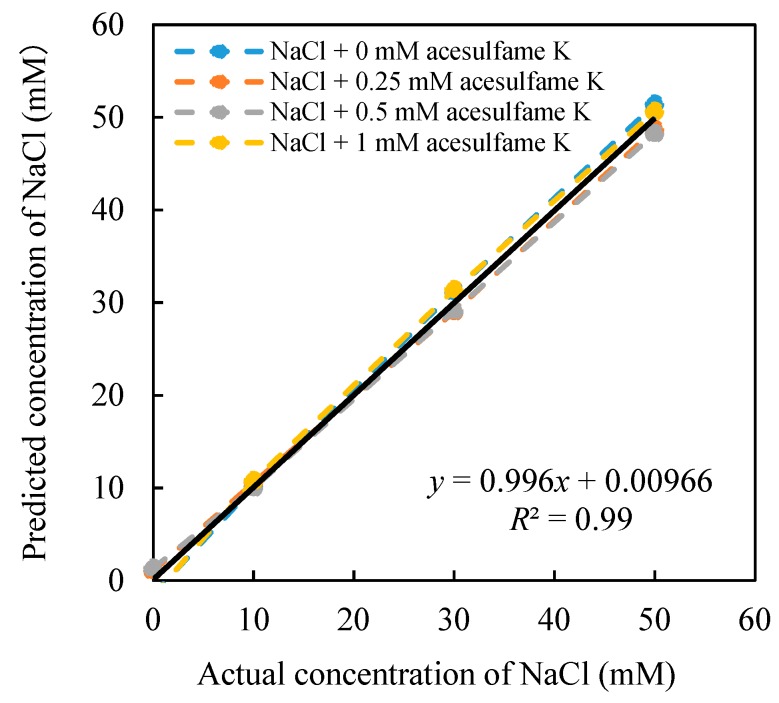
Relationship between the estimated concentration and the actual concentration of NaCl.

After the 16 sets of relative value data are substituted into the constructed model, all the coefficients can be obtained. The model was used to predict the concentration of the acesulfame K, as shown in Figure 5. On the other hand, the concentration of NaCl was also predicted, as shown in Figure 6, and numerical constants of Equations (8) and (9) are shown as follows: *a* = −50.75 mV, *b* = −47.6 mV, *c* = −0.024 mM, *d* = 114.51 mM, *e* = −16.92 mV, *f* = −54.35 mV, *j* = 0.50 mM, *k* = −0.49 mM, *m* = −177.45 mM, *n* = 176.35 mM. Equations (8) and (9) can be expressed in Equations (10) and (11).

Concentration of acesulfame K:

(10) $$x=0.50\cdot \exp\left(\frac{F}{-50.75}\right)-0.49\cdot \exp\left(\frac{G}{-47.6}\right)-0.024\cdot \left(\exp\left(\frac{F-G}{-16.92}\right)-1\right),$$

Concentration of NaCl:

(11) $$y=-177.45\cdot \exp\left(\frac{F}{-50.75}\right)+176.35\cdot \exp\left(\frac{G}{-47.6}\right)+114.51\cdot \left(\exp\left(\frac{F-G}{-54.35}\right)-1\right),$$

Figure 5 shows the high correlation between the estimated concentration and the actual concentration (*R*^2^ = 0.996). When the concentration of NaCl changes from 0–50 mM, the concentration of acesulfame K can be well predicted from 0–1 mM. Meanwhile, when the NaCl concentration changes, it does not affect the prediction of acesulfame K concentration. At the same time, the NaCl concentration can also be estimated simultaneously (Figure 6). The concentration of NaCl (0–50 mM) can be well predicted at different concentrations of acesulfame K (0–1 mM). Therefore, the model we created in this study can effectively predict the concentration of acesulfame K at 0–1 mM in mixed solutions. This analysis model has proved to eliminate the influence of saltiness successfully.

With the use of both a sweetness sensor and saltiness sensor, the influence of saltiness substances could be eliminated by the regression analysis method. The concentration of acesulfame K can be estimated with high precision in the mixture of acesulfame K and NaCl. To provide an accurate and convenient method of sweetness evaluation for commercial beverages and foods in the future, we hope to evaluate the sweetness of different kinds of sweetener mixtures by a multivariate analytical model.

## 4. Conclusions

In our previous research, a taste sensor for negatively charged, high-potency sweeteners was developed. This sensor has good selectivity when using CPA values, but did not show enough sensitivity at a low concentration of sweeteners. On the other hand, while the relative value of the sweetness sensor shows higher sensitivity, it is affected by salty substances. Therefore, this study attempts to establish a method to solve the selectivity problem of sweetness sensors when using relative values. This makes it possible to use relative values to evaluate sweetness. That will be helpful in improving the accuracy of the evaluation of the sweetness of high-potency sweeteners in mixture solutions.

We established a method to evaluate sweetness in mixture solutions using relative values of both a sweetness sensor and saltiness sensor. Since the saltiness sensor was used at the same time as the sweetness sensor, the interference caused by salty substances can be removed. Therefore, the model we created in this study can effectively predict the concentration of acesulfame K at 0.2–0.7 mM in a mixed solution with salty substances and show a high correlation (*R*^2^ = 0.996). As a result, the evaluation accuracy of the sweetness is improved successfully using the relative value. Further studies are needed to adjust the model so that the evaluation method can be applied to evaluate the sweetness of different kinds of sweetener mixtures (e.g., saccharin Na and aspartame). In practical applications, this method still has some limitations. It is necessary to investigate interfering substances at first while the beverages and foods are complicated; the effects of interfering substances can be eliminated using pretreatment or multivariate analysis. And it is possible to use the relative value to get a higher sensitivity of evaluation.

## Figures and Tables

**Table 1:** Samples of different tastes (the concentration of basic taste samples is ten times the human threshold).

Taste	Taste Samples
Astringency	0.05 wt% Tannic acid
Saltiness	300 mM Sodium chloride
Bitterness	0.01 vol% Iso alpha acid
Sourness	3.0 mM Tartaric acid
Sweetness (+)	10 mM Aspartame
Sweetness (−)	0.5 mM, 5 mM Saccharin Na
Sweetness (−)	1 mM, 10 mM Acesulfame K
